# Are we braver today in using antidepressants perinatally?- own clinical experiences

**DOI:** 10.1192/j.eurpsy.2024.1667

**Published:** 2024-08-27

**Authors:** K. Matić, S. Vuk Pisk, E. Ivezić, V. Grošić, I. Filipčić

**Affiliations:** ^1^University Psychiatric Clinic Sveti Ivan, Zagreb; ^2^Faculty of Dental Medicine and Health, Osijek; ^3^ Faculty of Dental Medicine and Health; ^4^University of Zagreb School of medicine, Zagreb, Croatia

## Abstract

**Introduction:**

Affective disorders are among the most common mental health problems in women of reproductive age. A life-changing condition, such as pregnancy, may trigger or intensify symptoms of affective disorders, rather than pregnancy being a protective factor for the development of the disorder, as previously thought. Previous research indicates that 18% of women suffer from perinatal depression. One in 7-10 pregnant women and 1 in 5-8 midwives develops a depressive disorder, which is more than half a million women a year. Untreated perinatal depression has significant repercussions for both mother and child. Given that there are no controlled randomized studies during pregnancy, and the results of previous research on the harmfulness of the use of psychotropic drugs are contradictory, we need to nurture an individualized and integrative approach to the use of psychotropic drugs in pregnant women and in the postpartum period. The goal of this lecture is to point out the necessity of treating perinatal depressive disorder with an emphasis on the need to work on dilemmas and selected sources of information by pregnant women themselves, as well as health professionals. In the end, I must emphasize the importance of choosing an adequate psychopharmaceutical in that sensitive period for a woman, nurturing an individual approach as well as the latest knowledge.

**Objectives:**

The aim of this research is to examine the attitudes of psychiatrists, GP doctors, gynecologists and pregnant women about prescribing and taking pharmacotherapy during pregnancy.

**Methods:**

The research will be conducted at the psychiatry clinic, the gynecology clinic and in health centers through semi-structured questionnaires, which will be filled out by psychiatrists, gynecologists, doctors and pregnant women.

**Results:**

Preliminary results (given that the research is still ongoing) indicate that most psychiatrists avoid prescribing drugs during pregnancy, and if they decide to do so, then diazepam is prescribed. The views of gynecologists, family medicine doctors and pregnant women are still pending.

**Conclusions:**

This lecture aims to point out the factors contributing to the fear of prescribing psychotropic drugs perinatally, based on our own research, which included psychiatrists, gynecologists, family medicine doctors, and pregnant women.

**Disclosure of Interest:**

None Declared

